# Genetic Predictors of Poor Prognosis in Portuguese Patients with Juvenile Idiopathic Arthritis: Data from Reuma.pt

**DOI:** 10.1155/2015/706515

**Published:** 2015-10-04

**Authors:** Ana Filipa Mourão, Maria José Santos, Sílvia Mendonça, Filipa Oliveira-Ramos, Manuel Salgado, Paula Estanqueiro, José Melo-Gomes, Fernando Martins, Ana Lopes, Bruno Filipe Bettencourt, Jácome Bruges-Armas, José Costa, Carolina Furtado, Ricardo Figueira, Iva Brito, Jaime Branco, João Eurico Fonseca, Helena Canhão

**Affiliations:** ^1^Rheumatology Research Unit, Instituto de Medicina Molecular, 1649-028 Lisbon, Portugal; ^2^Rheumatology Department, Hospital Egas Moniz, Centro Hospitalar Lisboa Ocidental, 1349-019 Lisbon, Portugal; ^3^CEDOC, Faculdade de Ciências Medicas da Universidade Nova de Lisboa, 1150-190 Lisbon, Portugal; ^4^Rheumatology Department, Hospital Garcia de Orta, 2801-951 Almada, Portugal; ^5^Portuguese Society of Rheumatology, 1800-033 Lisbon, Portugal; ^6^Rheumatology Department, Lisbon Academic Medical Center, 1649-028 Lisbon, Portugal; ^7^Pediatrics Department, Centro Universitário Hospitalar de Coimbra, 3041-801 Coimbra, Portugal; ^8^Instituto Português de Reumatologia, 1601-901 Lisbon, Portugal; ^9^Institute for Molecular and Cell Biology (IBMC), University of Porto, 4150-180 Porto, Portugal; ^10^Hospital de Santo Espírito da Ilha Terceira, SEEBMO, Angra do Heroísmo, 9700-049 Açores, Portugal; ^11^Instituto de Investigação e Inovação em Saúde, Universidade do Porto, 4150-180 Porto, Portugal; ^12^Rheumatology Department, Hospital Conde de Bertiandos, ULSAM, 4990-041 Ponte de Lima, Portugal; ^13^Rheumatology Department, Hospital do Divino Espírito Santo, São Miguel, 9500-370 Açores, Portugal; ^14^Rheumatology Department, Hospital Dr. Nélio Mendonça, Funchal, 9004-514 Madeira, Portugal; ^15^Rheumatology Department, Hospital de São João, 4200-319 Porto, Portugal; ^16^Faculdade de Medicina da Universidade do Porto, 4200-450 Porto, Portugal

## Abstract

*Introduction.* This study aimed to assess the genetic determinants of poor outcome in Portuguese patients with juvenile idiopathic arthritis (JIA). *Methods.* Our study was conducted in Reuma.pt, the Rheumatic Diseases Portuguese Register, which includes patients with JIA. We collected prospectively patient and disease characteristics and a blood sample for DNA analysis. Poor prognosis was defined as CHAQ/HAQ >0.75 at the last visit and/or the treatment with biological therapy. A selected panel of single nucleotide polymorphisms (SNPs) associated with susceptibility was studied to verify if there was association with poor prognosis. *Results.* Of the 812 patients with JIA registered in Reuma.pt, 267 had a blood sample and registered information used to define “poor prognosis.” In univariate analysis, we found significant associations with poor prognosis for allele A of* TNFA1P3/20* rs6920220, allele G of* TRAF1/C5* rs3761847, and allele G of* PTPN2* rs7234029. In multivariate models, the associations with* TRAF1/C5* (1.96 [1.17–3.3]) remained significant at the 5% level, while* TNFA1P3/20* and* PTPN2* were no longer significant. Nevertheless, none of associations found was significant after the Bonferroni correction was applied. *Conclusion.* Our study does not confirm the association between a panel of selected SNP and poor prognosis in Portuguese patients with JIA.

## 1. Introduction

Juvenile idiopathic arthritis (JIA) is the most common childhood rheumatic disease [1]. Despite significant improvements in the management of children with JIA, the likelihood of long-term persistent disease activity remains high [2]. Published evidence demonstrates that clinical subtype, disease activity and duration, and response to treatment all influence the prognosis [3, 4]. In addition, diagnostic delay, severity and extension of arthritis at onset, symmetric disease, early hip or wrist involvement, involvement of cervical spine, the presence of rheumatoid factor (RF) or anticyclic citrullinated peptide, early age at onset, female gender, and family history of rheumatic disease were the best predictors of a poor outcome [3, 5–11]. However, in most studies of prognostic predictors in JIA, the authors are unanimous in concluding that there is considerable variability in results, making it harder to draw consistent conclusions [8].

Identifying earlier JIA worse prognosis cases is crucial to start appropriate treatment and to correctly inform patients and their parents. Much effort has already been done to elucidate prognosis predictors. Besides clinical factors, identification of genetic predictors of poor prognosis would be a significant contribution to the development of optimal treatment strategies for JIA.

Studies that evaluate nonclinical predictors, such as genetic or immunological parameters, hardly exist. Most of the genetic research aimed to identify variants that affect the risk of developing JIA or pathways modulating drug response in this disease. On the contrary, the goal of this study was to assess the genetic determinants of poor outcome in Portuguese patients with JIA. Our secondary objective was to find potential clinical predictors of poor prognosis.

## 2. Methods

### 2.1. Patient Population

Our study was conducted based on Reuma.pt, the Rheumatic Diseases Portuguese Register, which includes JIA patients treated with synthetic and biological Disease Modifying Antirheumatic Drugs (DMARDs) since June 2001. Patients registered up to December 2013 were included. The parent's consent and patient's assent (as appropriate) were obtained according to the declaration of Helsinki. The study was approved by local Ethics Committee. All patients fulfilled the ILAR criteria for the classification of JIA [12]. This study did not have any interference with patients' standard of care.

We analyzed the patients registered in Reuma.pt with the diagnosis of JIA, who had collected a blood sample for DNA analysis. The following data were collected at the time of the last visit to rheumatology clinics: gender, age, JIA subtype, disease duration, time until diagnosis (time since the beginning of the symptoms until the diagnosis of JIA), extra-articular manifestations of the disease, duration of therapy with DMARDs, corticosteroids and biological therapies, Childhood Health Assessment Questionnaire (CHAQ)/Health Assessment Questionnaire (HAQ) [13], patient's/parent's pain visual analogue scale (VAS), patient's/parent's disease global activity VAS, and physician's global disease activity VAS.

One of the barriers found in prognostic studies of JIA is that there is no universal definition of “poor prognosis.” We have chosen to integrate in our definition of “poor prognosis” two variables: one instrument that combines disease activity and damage (CHAQ), dichotomized in accordance with other studies [14–19], using 0.75 as the cut-off point, combined with “the need for biological therapy,” as a surrogate marker of disease severity and higher likelihood of a worse outcome. We have classified as patients in “need for biological therapy” all patients that were ever treated with biological agents for more than 3 months, due to articular or extra-articular manifestations of the disease. Thus, for the purpose of this study a patient was classified as having poor prognosis if CHAQ >0.75 and/or if the patient was ever treated with biological therapy for more than 3 months.

Genetic single nucleotide polymorphisms (SNPs) were studied to verify if there was any association with poor prognosis.

### 2.2. Genetic Analysis

The choice of SNP variants was based on information from previous studies of susceptibility and prognosis factors in JIA and included the following 32 SNPs of genes with a known function in the immune system:* PTPN22* rs2476601,* PTPRC* rs10919563,* TNFAIP3/A20* rs10499194,* TNFAIP3/A20* rs6920220,* TRAF1/C5* rs3761847,* ANGPT1* rs1010824,* ANGPT1* rs7151781,* AFF3* rs1160542,* AFF3* rs10865035,* CTLA4* rs3087243,* ERAP1/ARTS1* rs30187,* IL1* rs6712572,* IL1* rs2071374,* IL1* rs1688075,* IL10-1080GA* rs1800896,* IL10-819CT* rs1800871,* IL1R* rs12712122,* IL23R* rs11209026,* IL2-IL21* rs6822844,* IL2RA/CD25* rs2104286,* MIF-173CG* rs755622,* PTPN2* rs1893217,* PTPN2* rs7234029,* SLC26A2* rs1541915,* STAT4* rs3821236,* STAT4* rs7574865,* TNF*-*238* rs361525,* TNF*-*308* rs1800629,* VTCN1* rs10923223,* VTCN1* rs12046117,* WISP3* rs2280153, and* EYA4* rs17301249.

All samples were genotyped using Taqman SNP genotyping assays (Applied Biosystems, Foster City, USA) performed as described in the manufacturers' protocol. Genotyping reactions were carried out with an ABI 7500-fast thermocycler. The allele call was obtained by the AB software v2.0.5, by the analysis of allelic discrimination plots. SNPs with deviation from Hardy-Weinberg equilibrium (*P* < 0.05) or minor allele frequency (MAF) <1% were excluded from further analysis.

### 2.3. Statistical Analysis

We used the additive model to study the association between SNPs and poor prognosis, where homozygotes for the major allele were classified as zero, heterozygotes as 1, and homozygotes for the minor allele as 2. We report crude odds ratio (OR) based on a univariate logistic regression and adjusted OR from a multivariate model including significant clinical predictors. The following clinical variables were characterized: gender, disease category (classified into five groups including polyarticular JIA (RF negative, RF positive, and extended oligoarticular), persistent oligoarticular JIA, systemic arthritis, enthesitis-related arthritis (ERA), and psoriatic arthritis), time until diagnosis (years), age at disease onset, disease duration (years), duration of DMARD treatment (years), corticosteroid treatment (ever or never), patient's/parent's disease global activity VAS, physician's global disease activity VAS, and extra-articular manifestations (yes or no). Continuous variables were modelled as linear. All clinical variables crudely associated with poor prognosis (*P* < 0.20) were included in a multivariate model. Then backward selection was applied to retain the clinical variables most associated with the outcome, using a significance level of 5%. Due to small sample size for most of the disease categories we carried out the analysis using all JIA categories combined. The stratified analysis was only possible for the polyarticular categories (polyarticular RF positive, polyarticular RF negative, and extended oligoarticular JIA) with 116 patients.

There was missing data for some of the variables, as follows: age at disease onset (1.5%), age at diagnosis (1.9%), patient's/parent's VAS (4.9%), and physician VAS (9.7%).

Statistical significance was considered at the 5% level. After Bonferroni correction for the 32 SNPs analyzed, results were considered significant for *P* < 0.0016.

Statistical analysis was made in R version 2.15.3 [20].

## 3. Results

Twenty-one centers and 77 rheumatologists and pediatricians contributed with data to Reuma.pt. Of the 812 patients with JIA registered in Reuma.pt (mean age 19.9 ± 11.3 years old, 65% females, and mean age at JIA onset 6.9 ± 4.7 years old), 291 had a blood sample to perform the genetic analysis and, from those, 267 had registered information about CHAQ/HAQ and/or the need for biological therapy used to define “poor prognosis.” Of the 267 patients included, 85 had a poor prognosis, according to the definition: CHAQ/HAQ >0.75 and/or the treatment with biological therapy for more than 3 months. Nineteen patients had a CHAQ/HAQ >0.75 at the last appointment, 58 were treated with biological therapy, and 8 fulfilled both criteria.

Table 1 shows the distribution of the clinical characteristics of patients with and without poor prognosis.

### 3.1. Clinical Predictors of Poor Prognosis

Almost all the clinical variables, except gender, age at disease onset, and delay in diagnosis, were significantly different between the group of JIA patients with poor prognosis and the group who did not had poor prognosis (Table 1).

Clinical variables significantly associated with poor prognosis and included in the multivariate models were DMARD treatment (OR 1.17 [95% confidence interval 1.07–1.27]), higher physician VAS (1.03 [1.01–1.04]), and disease category. In particular, the persistent oligoarticular category had a much lower chance of worse prognosis (0.09 [0.04–0.22]) compared to the polyarticular category; ERA (0.44 [0.18–1.09]), systemic arthritis (1.11 [0.45–2.76]), and psoriatic arthritis (0.55 [0.16–1.94]) categories were not significantly different to the polyarticular group of JIA.

### 3.2. Genetic Predictors of Poor Prognosis

Crude and adjusted odds ratios for the association between studied SNPs and poor prognosis are shown in Table 2. In univariate analysis including all disease categories we found significant associations with poor prognosis for allele A of* TNFA1P3/20* rs6920220, allele G of* TRAF1/C5* rs3761847, and allele G of* PTPN2* rs7234029. In multivariate models adjusted for relevant clinical predictors (disease category, DMARD treatment, and physician VAS) the association for* TRAF1/C5* rs3761847 (1.96 [1.17–3.30]) remained significant at the 5% level while* TNFA1P3/20* rs6920220 (1.67 [0.98–2.83]) and* PTPN2* rs7234029 (1.75 [0.99–3.10]) were no longer significant.

In the univariate analysis for the polyarticular categories we found associations for allele A of* CTLA4* rs3087243 and allele G of* PTPN2* rs7234029. After adjusting for clinical factors the associations for* CTLA4* rs3087243 (2.90 [1.39–6.08]) and* PTPN2* rs7234029 (3.30 [1.48–7.37]) were still significant at the 5% level.

Nevertheless, none of associations found was significant after the Bonferroni correction (*P* < 0.0016).

## 4. Discussion

The aim of our study was to identify genetic and clinical predictors of poor outcome in JIA. In a Portuguese sample of patients with JIA, we have not found genetic associations with a poor outcome. Longer duration of DMARD treatment, higher physician VAS, and polyarticular categories of JIA had a significant association with poor prognosis.

A growing number of studies have been focused on susceptibility to JIA, including genome wide association studies [21]. However, studies on genetics of JIA outcomes are still scarce. In a recent systematic literature review of early predictors of prognosis in JIA [8], the authors concluded that demographic, clinical, and laboratory values were insufficient to predict the individual prognosis. The authors also pointed out that hardly any other potential predictors were evaluated, such as cytokine levels, cell characteristics, results of imaging obtained early in the disease course, or genetic evaluations, such as HLA and SNPs in genes with a known function in the immune system.

There are some examples of genetic research on JIA outcomes, including a study that suggests that the* MIF-173* polymorphism (*MIF-173∗*C allele) is a predictor of poor outcome in systemic-onset JIA [22], another study that found SNPs in the* IL6* gene associated with pain [14] and a correlation between* TGF-b1* gene codon 25 genotypes and early radiological damage [14], and, in the ERA subtype, a publication suggesting that the presence of* HLA-DRB1∗08* predicts failure to attain disease remission [23].

RA shares several clinical and pathological features with JIA and previous studies reported considerable overlap in genetic susceptibility loci for the two diseases [24–26]. JIA is a heterogeneous disease and genetic differences across the JIA categories and some category-specific effects have been identified [27, 28]. However, stratified analysis leads to small sample sizes for many of the categories. Larger cohorts of the ILAR categories are required to improve the power to detect any category-specific effects. We have stratified our analysis to investigate the polyarticular categories (polyarticular RF positive, polyarticular RF negative, and extended oligoarticular) which are the largest category in our sample.

We have found an association between a variant in the* TRAF1/C5* locus and poor prognosis in Portuguese with JIA regardless of the disease category. Only in the polyarticular category of JIA did we find an association between 2 variants in the* CTLA4* and* PTPN2* loci and a poor outcome. Nevertheless, none of the associations found was significant after the Bonferroni correction was applied (*P* < 0.0016).

The analysis of the clinical variables identified a number of parameters associated with poor outcome. Patients with a poor prognosis were more likely to have polyarticular categories of JIA (polyarticular RF negative, polyarticular RF positive, and extended oligoarticular), to be on treatment with DMARDs for a longer period, and to have higher values of physician VAS at the last visit. Additionally, patients with a poor prognosis were less likely to have persistent oligoarticular JIA. Our results are in accordance with other studies that revealed that children with persistent oligoarticular JIA have a substantially better outcome than those with either systemic or polyarticular JIA, as measured by attaining remission, degree of disability, and structural damage [8, 10, 11].

There are some limitations in our study, namely, the definition used to determine poor prognosis. There is no universal definition of “poor prognosis” in patients with JIA. We have chosen to integrate in our definition of “poor prognosis” two variables: (1) an instrument that combines disease activity and damage (CHAQ/HAQ); (2) the need for biological treatment, because patients that do not respond to conventional DMARDs, namely, methotrexate, have a higher chance of a poor outcome. Regarding this last point, our study included patients at different phases of their disease and we are aware that the access to biological therapy could not have been the same for all patients, leading to a selection bias. In addition, some patients could have started biological therapy mainly for extra-articular manifestations of the disease (e.g., uveitis) and not due to joint disease. This could also have potentially confounded our results.

Another limitation of our study is the problem of multiple comparisons: our results may simply be attributable to chance. The sample size in our cohort was too small to adequately test replication and a further study in a larger cohort is still required in order to confirm or refute our findings.

## 5. Conclusions

In summary, our study does not confirm the association between a panel of selected SNPs and poor prognosis in Portuguese patients with JIA. A search for additional genetic variants is required. Moreover, combination of genetic factors together with environmental exposures should also be considered. Further studies, in different populations of JIA patients, should be performed to replicate these findings.

## Figures and Tables

**Table 1:** Distribution of the clinical characteristics of patients with and without poor prognosis.

Variable	Total	Patients with poor prognosis	Patients without poor prognosis	*P* value
Number	267	85	182	
Female gender *n* (%)	171 (64)	60 (22.59)	111 (41.6)	0.166
JIA categories (%):				
Polyarticular RF negative	48 (18)	19 (39.6)	29 (60.4)	<0.001
Polyarticular RF positive	25 (9.4)	19 (76)	6 (24)	
Extended oligoarticular	43 (16.1)	17 (39.5)	26 (60.5)	
Persistent oligoarticular	89 (33.3)	7 (7.9)	82 (92.1)	
Systemic	22 (8.2)	11 (50)	11 (50)	
Enthesitis-related arthritis	28 (10.5)	8 (28.6)	20 (71.4)	
Psoriatic arthritis	12 (4.5)	4 (33.3)	8 (66.7)	
Age at disease onset (median (IQR))	5.3 (2.2–9.7)	6.6 (3.1–11.6)	4.8 (2.1–8.8)	0.056
Age at diagnosis (median (IQR))	6.6 (2.8–11.6)	8.7 (3.4–13.7)	5.7 (2.5–10.6)	0.013
Time until diagnosis (median (IQR))	0.33 (1.14–1.00)	0.50 (0.17–1.0)	0.26 (0.14–0.88)	0.130
Age at last visit (median (IQR))	14.3 (8.9–18.3)	16.9 (13.1–24.1)	12.7 (6.9–13.3)	<0.001
Disease duration (median (IQR))	6.4 (3.1–12.0)	10.4 (5.2–16.0)	4.9 (2.3–10.4)	<0.001
CHAQ/HAQ (median (IQR))	0 (0–0.25)	0.25 (0-1)	0 (0–0.13)	<0.001
Patient's/parent's VAS (median (IQR))	5 (0–30)	10 (0–50)	0 (0–30)	0.017
Physician VAS (median (IQR))	0 (0–20)	10 (0–35)	0 (0–11.3)	<0.001
Extra-articular manifestations	98	41	57	0.011
Duration of DMARD use (median (IQR))	2.37 (0–5.8)	5.46 (3.02–9.54)	1.43 (0–4.02)	<0.001
Corticosteroid use (Y/N)	124	52	68	<0.001

F: female; M: male; JIA: juvenile idiopathic arthritis; RF: rheumatoid factor; IQR: interquartile range; CHAQ: Childhood Health Assessment Questionnaire; HAQ: Health Assessment Questionnaire; SD: standard deviation; VAS: visual analogue scale; DMARD: Disease Modifying Antirheumatic Drug; Y: yes; N: no. *P* values < 0.05 were considered statistically significant. Note: *P* values are from Pearson's chi-squared or Mann-Whitney tests, as appropriate.

**Table 2:** Crude and adjusted *odds ratio *for the association between single nucleotide polymorphisms and poor prognosis.

	Minor allele	Crude		Adjusted^†^	
		OR (95% CI)	*P* value	OR (95% CI)	*P* value
All categories					
*TNFA1P3/20* rs6920220 A/G	A	1.53 (1.01–2.33)	0.0436	1.67 (0.98–2.83)	0.0579
*TRAF1*/C5 rs3761847 A/G	G	1.49 (1.00–2.21)	0.0491	1.96 (1.17–3.3)	0.0110
*PTPN2* rs7234029 A/G	G	1.86 (1.17–2.95)	0.0085	1.75 (0.99–3.1)	0.0540
Polyarticular categories					
*CTLA4* rs3087243 A/G	A	1.98 (1.14–3.45)	0.0153	2.9 (1.39–6.08)	0.0047
*PTPN2* rs7234029 A/G	G	3.08 (1.53–6.19)	0.0016	3.3 (1.48–7.37)	0.0035

^†^Clinical covariates included disease category, DMARD treatment, and physician VAS. Disease category was omitted from the model for the polyarticular categories of JIA.

OR: odds ratio; SNPs: single nucleotide polymorphisms. *P* values < 0.05 were considered statistically significant.

## Abbreviations

JIA: Juvenile idiopathic arthritis
DMARD: Disease Modifying Antirheumatic Drug
CHAQ: Childhood Health Assessment Questionnaire
SNP: Single nucleotide polymorphism
VAS: Visual analogue scale
MAF: Minor allele frequency
OR: Odds ratio
RF: Rheumatoid factor
ERA: Enthesitis-related arthritis.
